# Retrieval-Induced Forgetting in the Feigning Amnesia for a Crime Paradigm

**DOI:** 10.3389/fpsyg.2019.00928

**Published:** 2019-04-26

**Authors:** Ivan Mangiulli, Kim van Oorsouw, Antonietta Curci, Marko Jelicic

**Affiliations:** ^1^Forensic Psychology Section, Faculty of Psychology and Neuroscience, Maastricht University, Maastricht, Netherlands; ^2^Department of Education, Psychology, Communication, University of Bari Aldo Moro, Bari, Italy

**Keywords:** feigning amnesia, retrieval-induced forgetting, inhibition, malingering, memory errors

## Abstract

Previous studies demonstrated that, when asked to honestly provide information about a mock crime, former feigners performed worse than those who were requested to confess to this event. Thus, feigning amnesia for a mock crime undermined genuine memory for the same experience. In the present study, we examined whether retrieval-induced forgetting (RIF) underlies this memory-undermining effect. After watching a mock crime, participants had to feign amnesia or confess to having committed that crime. Feigners were given retrieval practice instructions (i.e., retrieval-practice group) or no further instructions (i.e., control group). Immediately and 1 day later, all participants had to genuinely report what they remembered about the crime. Although simulators in the retrieval-practice group recalled the largest amount of information as a positive consequence of retrieval, the ratio for crucial crime-related details was lower than that exhibited by both simulators who were given no instructions and confessors. These findings suggest that RIF might play a role in forgetting critical information in claims of crime-related amnesia. Theoretical and practical implications will be discussed.

## Introduction

Many offenders feign amnesia for serious crimes (Cima et al., 2002; Christianson and Merckelbach, 2004; Pyszora et al., 2014; Jelicic and Merckelbach, 2015; Jelicic, 2018). Although crime-related amnesia does not lead to incompetency to stand trial judgment, oftentimes defendants adopt this deceptive strategy to obstruct the trial proceedings (Tysse, 2005; Tysse and Hafemeister, 2006). Of importance, there is reason to believe that perpetrators who falsely claimed amnesia for the crime may later encounter actual problems in retrieving crime-related information. The memory-undermining effect of feigned amnesia has been observed in several studies (e.g., Christianson and Bylin, 1999; Bylin and Christianson, 2002; Van Oorsouw and Merckelbach, 2004, 2006; Mangiulli et al., 2018b; Romeo et al., 2018; Mangiulli et al., in press). Compared with those who were instructed to confess to a mock crime, participants who were previously asked to feign amnesia for this event exhibit poorer memory performance when they are subsequently requested to give up their role as feigner. Moreover, because feigning participants tend to comply with their instructions by omitting, distorting and introducing new information on the initial memory test (Bylin, 2002; Van Oorsouw and Merckelbach, 2006), simulating amnesia can also lead to both omission and commission errors on the final recall test (Van Oorsouw and Giesbrecht, 2008; Mangiulli et al., 2018b; Mangiulli et al., in press). One could argue, indeed, that simulating amnesia might be accomplished in at least two manners – by withholding or omitting information vs. by distorting and introducing new information (Bylin and Christianson, 2002; Mangiulli et al., 2018b; Otgaar and Baker, 2018). This avenue resembles what Bylin and Christianson (2002) have already tried to investigate. Participants were instructed to either confess to a traffic crime or feign amnesia by withholding or distorting information surrounding the event. This study showed that participants instructed to feign amnesia by omitting information recollected fewer correct details than those who were instructed to genuinely account for the crime (i.e., confessor). Yet, no significant differences were found between the two simulator groups or between feigners instructed to distort information and participants asked to confess to the crime. However, Bylin and Christianson (2002) used a small number of participants who decided themselves which crime-related information to omit and to retrieve, which might have obscured differences between conditions.

Several explanations were given with respect to the memory-undermining effect due to feigned amnesia claims (see Van Oorsouw and Merckelbach, 2006). For instance, Christianson and Bylin (1999; see also Bylin and Christianson, 2002) suggested that among those who feign amnesia for a crime, some tend to omit crucial crime-related information (e.g., how the crime occurred) but do report unimportant elements of the offense (e.g., the location of the crime). When feigners use this specific deceptive strategy, it may be the case that some details of the mock crime would be strengthened in simulators’ memory, while other details would be weakened, leading to poorer recall of some elements of the crime over time (e.g., Christianson and Bylin, 1999; Bylin and Christianson, 2002; Van Oorsouw and Merckelbach, 2004). According to some scholars (e.g., Christianson and Bylin, 1999; Bylin and Christianson, 2002), the simulating amnesia effect might be the result of retrieval-induced forgetting (RIF, Anderson et al., 1994;Levy and Anderson, 2002; Anderson, 2003).

Retrieval-induced forgetting is a memory phenomenon that occurs when selective retrieval of specific memories leads to forgetting of other related memories. It has been demonstrated by research using the retrieval-practice procedure (Anderson et al., 1994). To start with, participants are given a set of category-item pairs (e.g., fruit-banana, drink-gin). During the retrieval-practice phase, participants are requested to retrieve half of the studied items from half of the categories, triggered by cues to facilitate the recollection (e.g., fruit-ba____). After a distractor task, participants are tested on their ability to recall all the previously encoded items. Usually, participants are better able to recall retrieved items from a practiced category (e.g., fruit-banana; Rp + items) than both un-retrieved items from a practiced category (e.g., fruit-apple; Rp - items) and un-practiced items from an un-practiced category (e.g., drink-gin; Nrp items). This pattern is known as a positive consequence of retrieval, namely called *facilitation effect* (Roediger and Karpicke, 2006; Roediger and Butler, 2011). However, a second pattern is also observed: Rp - items are recalled worse than Nrp items, indicating reduced recalling of un-practiced items from the practiced category, also referred to as *retrieval-induced forgetting* (Anderson et al., 1994; Anderson, 2003).

By and large, according to the inhibition-based forgetting theory, the RIF effect is due to an inhibitory mechanism which supports selective retrieval by suppressing the conflict from related memories (e.g., Levy and Anderson, 2002; Anderson, 2003). From this perspective, the inhibition of un-practiced items from a practiced category (Rp -) produces the RIF effect during the retrieval-practice phase. However, theoretical implications regarding the effects of delay on RIF are still disputed. While some researchers argued that RIF reveals a temporary or transient reduction in the accessibility of items in memory (MacLeod and Hulbert, 2011; Raaijmakers and Jakab, 2013), other scholars argued that, to some degree, inhibition might have persisting consequences (Anderson, 2003; Storm et al., 2012). Note that the RIF effect has been extensively investigated and demonstrated within the area of eyewitness memory (e.g., Shaw et al., 1995; MacLeod, 2002; Migueles and Garcia-Bajos, 2007; Garcia-Bajos et al., 2009; Camp et al., 2012; Pica et al., 2015). Specifically, the impact of repeated questioning of a witness has been explored. In the initial crime-viewing phase, participants are shown a series of slides depicting several items belonging to two different categories – typically a set of category-item pairs of stolen objects and suspects’ characteristics – and asked to memorize all the items. During the interrogation phase, participants are questioned about half of the items of one category. When later asked to recall items from both categories, a RIF effect takes place, indicating forgetting of offender’s characteristics or crime scene (e.g., Shaw et al., 1995; MacLeod, 2002; Saunders and MacLeod, 2006; Migueles and Garcia-Bajos, 2007; Pica et al., 2015).

In real-life situations some defendants might be inclined to selectively retrieve, and omit, specific actual information about the crime in an attempt to be consistent with their claims of memory loss. While omitting important crime details, defendants might be more prompted to forgetting *actual* crucial information of the crime later on, rather than when they come up with a self-generated version of the crime, wherein they distort or introduce new fabricated information surrounding the criminal experience (Van Oorsouw and Giesbrecht, 2008; Mangiulli et al., in press). Germane to this, research has shown that individuals are able to remember and distinguish the content of self-generated information over time (Chrobak and Zaragoza, 2008, 2012; Ackil and Zaragoza, 2011; Mangiulli et al., 2018a). Therefore, a narrowed strategy (i.e., selectively retrieving and omitting just some actual details of the crime) as compared with a broader way of feigning amnesia (i.e., distorting and fabricating new information of the event; Van Oorsouw and Merckelbach, 2006), might lead offenders to forgetting crucial information surrounding the criminal act. Following this line, RIF could play a part in the memory-undermining effect of feigning amnesia.

### Overview and Hypotheses

In the current study, we sought to determine whether RIF might explain the simulating amnesia effect by applying the retrieval-practice procedure to the feigning amnesia paradigm. That is, by requesting simulating participants to adopt a narrowed strategy similar to the classical RIF procedure (e.g., Anderson et al., 1994), we aimed to investigate whether offenders’ memory impairments after feigned crime-related amnesia claims might be due to RIF. We exposed participants to a violent mock crime video (target event). Next, we requested participants to either feign amnesia by retrieving some actual information pertaining to the crime and simultaneously omitting other (i.e., practiced and un-practiced information, respectively; simulator retrieval-practice group), or feign amnesia without giving any specific information about how to do so (i.e., simulator control group). At the same time, we also involved a group of participants instructed to give an honest account of the mock crime (i.e., confessor group). Immediately after having feigned their memory loss for the mock crime, simulators were asked to genuinely recall the target event. Finally, after 1-day delay, we requested all groups to recall as much information as possible about the mock crime. We predicted that simulators in the retrieval-practice group would perform better for practiced and worse for un-practiced information, compared with those in the simulator control group both immediately and after 1 day delay (RIF effect, Anderson, 2003; Storm et al., 2012; hypothesis 1). Also, we expected that feigning amnesia would undermine memory for the mock crime, meaning that both simulator groups would recall a lower amount of correct information (i.e., practiced and un-practiced information) than confessor group on the final recall tests (hypothesis 2). Moreover, we anticipated both simulator groups to report more errors than confessors during both immediate and delayed tests (hypothesis 3).

## Materials and Methods

### Participants and Design

The current study was approved by the standing Ethical Committee of the Faculty of Psychology and Neuroscience, Maastricht University (ERCPN application – 180_01_06_2017). Using G^∗^Power (Faul et al., 2007), an *a priori* power analysis with power of 0.80 and a predicted medium effect size (*f* = 0.30) indicated that a sample of 111 participants was needed. A total of 120 students (74% women; *M*_age_ = 21.21, *SD* = 2.83) was recruited using SONA software and online advertising (e.g., Facebook targeting). They were randomly assigned to the three groups – simulators in the retrieval-practice (simulator RP; *N* = 40), simulator controls (*N* = 40), and confessors (*N* = 40). After participating, each person was rewarded with a course credit or a 10-euro voucher. The study adopted a 3 × 2 mixed model design with group (simulator RP vs. simulator controls vs. confessors) as between-subjects variable, and memory test (immediate vs. after 1 day) as a within-subjects repeated measure variable. The dependent variable was the proportion of correct crime-related information reported in the free recall tests (i.e., information type: Rp + and Rp -). Furthermore, we also calculated errors generated during each memory test.

### Measures

#### The Positive and Negative Affect Schedule-Trait and State (PANAS–T and –S; Watson et al., 1988)

The scales require participants to rate on twenty 5-point items how they experience different emotional states along two dimensions, matching to Positive Affect (PA) and Negative Affect (NA). For both PANAS–T^1^ and –S item scores were summed up. The PA–T scale (α = 0.72) indicates the individual positive level of emotions generally felt by people, while in contrast the NA–T scale (α = 0.88) indicates the individual general dimension of aversive affect and distress. The PA–S (α = 0.85) and the NA–S (α = 0.91) scales reflect affective experiences of an individual at that precise moment.

#### Mock Crime Event

Accompanied by background music, a mock crime video recorded in *point of view* (*pov*) perspective^2^ was employed as a crime stimulus for this study. The video (about 3 min) showed a seemingly normal day in which a person comes home after a hard day’s work. After having dinner at his/her flat, the person decides to go to the inner city for some drinks. In the restroom of the last club, the offender has a violent fight with another person. The scene ends with the strangling of the victim.

### Procedure

#### Session 1

After signing the informed consent, PANAS–T and –S were assessed as a baseline measure for the participants’ emotional state prior to being subject to the mock crime video. Next, participants were invited to pay attention to the mock crime and they were requested to identify themselves with the character that performed actions in the video (the offender). PANAS–S was also administered a second time, immediately after the mock crime video, to examine the affective impact of the stimulus material. A 10 min distractor task followed the video presentation during which all participants played a computer game (e.g., Tetris). After this distractor task, participants were asked to imagine that they had been arrested on suspicion of murder and that, in a few days’ time, they would have to stand trial. Of importance, we adapted the RIF procedure (e.g., Migueles and Garcia-Bajos, 2007; Garcia-Bajos et al., 2009) in such a way that it could be used in the feigning amnesia paradigm. Similar to previous studies (e.g., Christianson and Bylin, 1999; Van Oorsouw and Merckelbach, 2004; Mangiulli et al., 2018b; Mangiulli et al., in press), we asked participants to report their statements through a free recall memory test in accordance with one of the following conditions.

After watching the mock crime video, in order to evade responsibility for the criminal act, simulators in the retrieval-practice group (simulator RP) were asked to study 20 actual pieces of the mock crime video (Rp +) which covered the sequence of the event from the beginning to the end while, at the same time, omitting crucial details pertaining to the mock crime (Rp -) (see Appendix A^3^). Next, they were given a cued recall task to test their memory for the 20 pieces of information they had studied (see Appendix B). After a 5 min distractor task (i.e., Tetris), we invited simulators RP to account for the crime (i.e., simulation phase) by retrieving and writing down the information they previously studied and practiced (Rp +), thereby inducing feigned memory loss for all the other crucial crime-related information (Rp -). Finally, after another 5 min distractor task, participants were given a free recall test, wherein simulators were requested to cooperate with the police by giving up their role as feigner and genuinely report all they could remember about the mock crime (i.e., immediate recall phase).

Still with the purpose of evading responsibility for the crime, participants in the simulator control group were asked to simulate memory loss after being exposed to the mock crime video. Contrary to simulators RP, during the simulation phase, simulators in the control group did not receive any specific instruction regarding which strategy to use to feign amnesia for the mock crime. Hence, they were free to omit, distort, and/or even report other information, pretending to have any difficulty in remembering what occurred. After a 10 min distractor task, also participants in this group were asked to cooperate with the police and honestly report about the mock crime act by recollecting as many details as possible (i.e., immediate recall).

Finally, in contrast with both simulator groups, after viewing the mock crime and performing the same 10 min distractor task, participants in the confessor group were directly given a recall test, meaning that they were not involved in the simulation phase. Confessors, thus, were instructed to collaborate with the police and admit their guilt by genuinely reporting as many details as possible about the mock crime. Finally, once all participants (simulator RP vs. simulator controls vs. confessors) completed the immediate recall, they were scheduled for a second session the following day.

#### Session 2

After a 24-h delay, all participants (simulator RP vs. simulator controls vs. confessors) were given a free recall test and, again, were asked to provide as much crime-related information as possible (i.e., after 1 day recall). Next, participants rated their ability to identify themselves with the offender^4^ on a 5-point scale anchoring from 0 (“*Not at all*”) to 4 (“*At all*”). Finally, participants were individually thanked and debriefed.

### Memory Recall Scoring

Note that, in the current study, the simulator control group and confessor group were not subjected to the retrieval practice manipulation and their memory performance served as a baseline to ascertain both facilitation and RIF effects due to the retrieval practice procedure (e.g., Nrp condition; Shaw et al., 1995; Migueles and Garcia-Bajos, 2007). Indeed, although only simulator RP were subjected to the retrieval practice manipulation, we employed the following scoring system for each participant’s report. Specifically, participants scored 1 point (maximum = 40) for each correct unit of information provided (i.e., both Rp +: “*I got in my green car*,” and Rp -: “*I strangled the victim*”). Following previous studies (e.g., MacLeod, 2002; Migueles and Garcia-Bajos, 2007), both Rp + and Rp - scores were transformed into proportions (range = 0–1) by dividing the amount of correct information provided by the maximum obtainable score. Additionally, the number of distortions (e.g., “*The victim pushed me against the wall*”) and commissions (i.e., the introduction of new information that was not displayed in the video: “*The victim had a knife*”) were calculated and collapsed into one score (i.e., errors). The first author and a research assistant, who was blind to the hypotheses and design of the study, scored participants’ free recall reports. The Interclass Correlation Coefficient (ICC) average measure for both Rp + and Rp - information was 0.97 and 0.81 (*p*_s_ < 0.001). The ICC for errors was 0.83 (*p* < 0.001).

## Results

### Affective Impact of the Mock Crime Event

To evaluate the affective impact of the mock crime on participants, a 3 × 2 mixed model ANOVA with group (simulator RP vs. simulator controls vs. confessors) as a between-subjects factor and pre-post mock crime viewing (pre-mock crime vs. post-mock crime) as a within-subjects factor was conducted. The main effects of the pre-post mock crime viewing for both PA-S and NA-S scores, *F*(1,117) = 67.24, *p* < 0.001, $\eta_{p}^{2}$ = 0.36, and *F*(1,117) = 116.61, *p* < 0.001, $\eta_{p}^{2}$ = 0.49, revealed that the mock crime event had an affective impact on participants by increasing their negative and reducing their positive mood state.

### Manipulation Check on Simulating Participants’ Instructions

To assess whether both simulator groups properly complied with their instructions, correct free recall scores (i.e., information type: Rp + and Rp -) were summed and entered in a 2 × 3 mixed model ANOVA with group (simulator RP vs. simulator controls) as a between-subjects factor and memory test (simulation vs. immediate vs. after 1 day) as a within-subjects factor. This analysis revealed significant main effects of group and memory test, *F*(1,77) = 117.26, *p* < 0.001, $\eta_{p}^{2}$ = 0.60, and *F*(1,77) = 187.66, *p* < 0.001, $\eta_{p}^{2}$ = 0.71. These main effects were qualified by a significant group by memory test interaction, *F*(1,77) = 22.75, *p* < 0.001, $\eta_{p}^{2}$ = 0.23, indicating that during the simulation phase participants in the simulator retrieval-practice group reported more correct information than those in the simulator control group, *t*(77) = 20, *p* = 0.001, *d* = 1.15. However, this effect was due to the retrieval-practice instruction since the retrieval rate for Rp + information in the simulator retrieval-practice group was 99% (*SD* = 0.03), meaning that our manipulation was successful. Indeed, the amount of correct information recollected over time increased in the retrieval-practice group, *t*(38) = 5.69, *p* < 0.001, *d* = 0.93, and *t*(38) = 7.30, *p* < 0.001, *d* = 1.21. Equally, as a result of the instruction given, participants in the simulator control group reported more correct information at both immediate and delayed memory test than during the simulation phase, *t*(39) = 11.13, *p* < 0.001, *d* = 1.99, and *t*(39) = 11.73, *p* < 0.001, *d* = 1.75.

### Facilitation Effect

Following previous research (e.g., Migueles and Garcia-Bajos, 2007), practiced information (Rp +) from the simulator retrieval-practice group and un-practiced information (Rp -) from the simulator control group were compared to ascertain the facilitation effect due to retrieval-practice. A 2 × 2 mixed model ANOVA with group (simulator RP vs. simulator controls) as a between-subjects factor, and memory test (immediate vs. after 1 day) as a within-subjects factor was conducted. This analysis highlighted the main effect of group was found to be significant, while the main effect of memory test was not, *F*(1,78) = 47.40, *p* < 0.001, $\eta_{p}^{2}$ = 0.38, and *F*(1,78) = 1.65, *p* = 0.20. A significant group by memory test interaction effect, *F*(1,78) = 4.67, *p* = 0.03, $\eta_{p}^{2}$ = 0.06, indicated that the retrieval-practice produced the facilitation effect (see Table 1). This means that participants in the simulator retrieval-practice group significantly disclosed more Rp + information than un-practiced information reported by those in the simulator control group at both immediate and after 1 day memory tests, *t*(78) = 5.18, *p* < 0.001, *d* = 2.63, and *t*(78) = 7.58, *p* < 0.001, *d* = 2.80, in accordance with our prediction^5^ (hypothesis 1). Furthermore, the facilitation effect slightly increased from the immediate to the 24-h delayed memory test, *t*(39) = 2.90, *p* < 0.001, *d* = 0.47.

**Table 1:** Mean proportions of practiced and un-practiced information by simulator groups, and retrieval-practice effects at both immediate and delayed memory tests.

	Simulators retrieval practice			Facilitation	RIF
				
	Rp +	Rp -	Control	(Rp +) – Control	(Rp -) – control
Immediate	0.63 (0.14)	0.34 (0.13)	0.46 (0.16)	0.17 (0.22)	-0.12 (0.23)
After a day	0.68 (0.12)	0.34 (0.14)	0.45 (0.15)	0.23 (0.17)	-0.11 (0.22)


*Rp + and Rp - information from the simulator retrieval-practice group are shown. Control displays un-practiced information from simulator control group. Standard deviations are presented between parentheses.*

### Retrieval-Induced Forgetting Effect

Un-practiced information from both simulator groups were analyzed to verify whether retrieval-practice caused a retrieval-induced forgetting effect (e.g., Migueles and Garcia-Bajos, 2007). A 2 × 2 mixed model ANOVA was performed with group (simulator RP vs. simulator controls) as a between-subjects factor and memory test (immediate vs. after 1 day) as a within-subjects factor. This analysis revealed only the main effect of group, *F*(1,78) = 14.18, *p* < 0.001, $\eta_{p}^{2}$ = 0.15, meaning that collapsing the data of both immediate and after 1 day recalls simulators in the retrieval-practice group (*M* = 0.34, *SD* = 0.12) reported less un-practiced information than simulator control group (*M* = 0.45, *SD* = 0.11), *t*(78) = -3.76, *p* < 0.001, *d* = 0.95, in line with our expectation^6^ (hypothesis 1). No other main or interaction effects were found to be significant, *F*_s_(1,78) < 0.38, *p* > 0.59.

### Free Recall – Correctness Scores

In order to ascertain differences between groups on the total amount of correct crime-related information provided (i.e., information type: Rp + and Rp -), a 3 × 2 mixed model ANOVA was performed with group (simulator RP vs. simulator controls vs. confessors) as a between-subjects factor and memory test (immediate vs. after 1 day) as a within-subjects factor. The main effect of group reached significance, *F*(2,177) = 19.88, *p* < 0.001, $\eta_{p}^{2}$ = 0.25. Partially supporting our hypothesis (hypothesis 2), this analysis revealed that only simulator control group (*M* = 0.34, *SD* = 0.13) disclosed less correct information than confessor group (*M* = 0.46, *SD* = 0.13), *p* < 0.001, *95% CI* (-0.18, -0.05), *d* = 0.92, while no significant difference was observed between simulators in the retrieval practice group (*M* = 0.50, *SD* = 0.09) and confessors, *p* = 0.46, *95% CI* (-0.02, 0.10), *d* = 0.35. Furthermore, participants in the simulator retrieval-practice group outperformed those in the simulator control group, *p* < 0.001, *95% CI* (0.09, 0.22), *d* = 1.43. No other main or interaction effects reached significance,*F*_s_(2,177) < 1.26, *p* > 0.29.

Given the unexpected pattern of findings described above, we conducted further analyses on the ratio rates for un-practiced information (i.e., crucial information pertaining to the crime) enclosed in participants’ total correct free recall scores. We calculated the ratio for un-practiced information (i.e., crucial information pertaining to the crime) by dividing the amount of Rp - information by the total amount of information [Rp -/(Rp +) + (Rp -)]. The ratio for Rp - information was entered in a 3 × 2 mixed model ANOVA with group (simulator RP vs. simulator controls vs. confessors) as a between-subjects factor and memory test (immediate vs. after 1 day) as a within-subjects factor. The main effect of condition *F*(1,177) = 133.18, *p* < 0.001, $\eta_{p}^{2}$ = 0.70, indicated that both confessors (61%, *SD* = 0.11) and participants in the simulators control group (69%, *SD* = 0.13) provided significantly more un-practiced information than simulators in the retrieval-practice group (34%, *SD* = 0.09), *p* < 0.001, *95% CI* (-0.22, 0.33), *d* = 2.68, and *p* < 0.001, *95% CI* (0.29, 0.40), *d* = 3.13, while participants in the simulator control group recollected slightly more crucial details of crime than those in the confessor group, *p* = 0.005, *95% CI* (0.02, 0.13), *d* = 0.66. These findings suggest that, although simulators in the retrieval-practice group recollected a remarkable number of details pertaining to the crime, the prevalence of crucial crime-related information in the free recall of those participants was significantly lower than that in the other two groups (see Table 2). No other main or interaction effects were found,*F*_s_(2,177) < 0.545, *p* > 0.58.

**Table 2:** Total correct proportions [i.e., (Rp +) + (Rp -)] and corresponding information type ratios [i.e., Rp +/(Rp +) + (Rp -), and Rp -/(Rp +) + (Rp -)] reported by each group during the three memory tests (simulation vs. immediate vs. after 1 day).

	Simulators retrieval practice			Simulators control			Confessors		
			
	Rp +		Rp -	Rp +		Rp -	Rp +		Rp -
Simulation		**0.41 (0.06)**			**0.11 (0.06)**				
	99% (0.03)		01% (0.02)	68% (0.21)		32% (0.22)			
Immediate		**0.49 (0.09)**			**0.35 (0.14)**			**0.46 (0.14)**	
	65% (0.10)		35% (0.11)	31% (0.13)		69% (0.13)	39% (0.10)		61% (0.10)
After 1 day		**0.51 (0.09)**			**0.39** **(0.13)**			**0.46 (0.15)**	
	67% (0.10)		33% (0.10)	32% (0.13)		68% (0.14)	38% (0.12)		62% (0.12)


*Although only simulators RP were subjected to the retrieval practice manipulation, Rp + and Rp - information is displayed to highlight the percentage of non-crucial (Rp +) and crucial (Rp -) details provided by participants in all the three experimental conditions. Total correct scores are displayed in bold text. Standard deviations are shown between parentheses.*

### Free Recall – Error Scores

A 2 × 3 mixed ANOVA with group (simulator RP vs. simulator controls) as a between-subjects factor and memory test (simulation vs. immediate vs. after 1 day) as a within-subjects factor, was run on the error scores (i.e., distortions and commissions). The main effect of group and the group by memory test interaction were found significant, *F*(1,73) = 34.62, *p* < 0.001, $\eta_{p}^{2}$ = 0.32, and *F*(2,146) = 25.62, *p* < 0.001, $\eta_{p}^{2}$ = 0.26, while there no significant effect of time, *F*(2,146) = 1.61, *p* = 0.20. This analysis revealed that simulators in the retrieval-practice group made more errors during both immediate and delayed memory tests compared to the simulation phase, *t*(36) = 6.26, *p* < 0.001, *d* = 1.01, and *t*(36) = 4.92, *p* < 0.001, *d* = 0.85. The simulator control group made fewer errors at both immediate and after 1 day memory tests compared to the simulation phase, *t*(37) = 3.97, *p* < 0.001, *d* = 0.52, and *t*(39) = 3.40, *p* = 0.002, *d* = 0.43 (see Table 3). Furthermore, during the simulation phase, this latter group made more errors than simulators in the retrieval-practice condition, *t*(75) = 7.30, *p* < 0.001,*d* = 1.69 (see Table 3).

**Table 3:** Total error scores provided by each group during the three memory tests (simulation vs. immediate vs. after 1 day).

	Simulators retrieval practice	Simulators control	Confessors
Simulation	0.32 (0.70)	6.17 (4.82)	
Immediate	1.82 (1.44)	3.68 (2.55)	2.54 (2.42)
After 1 day	2.30 (2.54)	3.87 (3.34)	2.65 (2.47)


*Errors are displayed in absolute numbers. Standard deviations are shown between parentheses.*

Finally, to compare differences between groups on the error scores, a 3 × 2 mixed ANOVA was conducted with group (simulator RP vs. simulator controls vs. confessors) as a between-subjects factor and memory test (immediate vs. after 1 day) as within-subjects factor. The main effect of condition was found to be significant, *F*(2,113) = 6.16, *p* = 0.003, $\eta_{p}^{2}$ = 0.10. Partially supporting our hypothesis (hypothesis 3), this analysis indicated that overall only participants in the simulator control group (*M* = 3.78, *SD* = 2.96) slightly made more errors than confessors (*M* = 2.52, *SD* = 2.37), while no significant differences were found between this latter group and participants in the simulator retrieval-practice condition (*M* = 2.06, *SD* = 2), *p* = 0.04, *95% CI* (0.02, 2.49), *d* = 0.47, and *p* = 1, *95% CI* (-0.76, 1.68), *d* = 0.20. Moreover, this analysis showed that simulators in the retrieval-practice group made fewer errors than participants in the simulator control group, *p* = 0.03, *95% CI* (0.49, 2.96), *d* = 0.68. No other main or interaction effects were found *F*_s_(1,113) < 1.09, *p* > 0.30.

## Discussion

In the current study we sought to examine whether the RIF effect might explain offenders’ memory detriments due to feigned crime-related amnesia claims. By forcing simulators to engage in retrieval practice, we predicted that those would better recall practiced information and more poorly recollect un-practiced information than participants in the simulator control group both immediately and after 1 day delay (hypothesis 1). Moreover, we hypothesized that both simulators groups would report fewer correct details (i.e., both practiced and un-practiced information) than confessors on the final recall tests (i.e., simulating amnesia effect; hypothesis 2). Finally, we expected that both simulator groups would report more errors than confessors during both tests (hypothesis 3).

With respect to our first hypothesis, retrieval-practice produced the expected facilitation and RIF effects. This pattern of results is consistent with the retrieval specificity principle (see for a review: Murayama et al., 2014). That is, the diminished recollection of un-practiced information is assumed to be caused by inhibition, which is likely to occur during the retrieval-practice. Anderson et al. (2000), for instance, observed that although expected RIF was exhibited when participants were asked to recall the practiced items (e.g., fruit-or____), their memory performance was unimpaired when they were requested to recall the category name (e.g., fr___-orange). Without prior retrieval, indeed, no inhibition of un-practiced information appears to be induced. One could argue that the very act of retrieving their feigned version of the crime – by firstly being involved in the cued task and secondly actively rehearsing the same Rp + information via free recall – led participants in the retrieval-practice group to inhibiting crucial crime-related memories. Moreover, the instructions to recall only Rp + information were likely to strengthen and consolidate simulators RP’s performance for those practiced items (e.g., Payne, 1987; Shaw et al., 1995), slightly increasing the positive effects of the retrieval-practice over time. However, simulators exposed to the retrieval-practice manipulation might have forgotten crucial-crime related information due to the strengthened recollection of practiced information first. Because we used a free recall task, indeed, we cannot rule out the contribution of output interference (Roediger, 1974; Tulving and Arbuckle, 1996). Output interference indicates that sometimes other explanations, rather than the inhibition-based mechanism, can account for the forgetting due to the retrieval practice (e.g., competition-based mechanism; Verde, 2012; Raaijmakers and Jakab, 2013). Still, even controlling for output interference, recent research has indicated that RIF effects still take place, for instance, in witness circumstances (e.g., Camp et al., 2012).

Relatedly, regarding our second hypothesis, the overall memory performance of participants in the simulator retrieval-practice group might have yielded a deceptive hypermnesia at the final test. That is, at first glance our findings may lead to the inference that prior retrieval of some crime information might have helped simulators in the retrieval-practice group to better remember the entire event and boost their memory over time. However, the percentage of crucial crime-related information (Rp -) remembered by those simulators was significantly lower than that exhibited by participants in the simulator control and confessor groups. In light of this, if we consider impairments of crucial information about the crime being the core of the feigning amnesia effect, our findings reflect the idea that RIF might play a part into feigners’ memory detriments in crime-related amnesia. Thus, extending recent research on the nature of retrieval-induced forgetting (e.g., MacLeod and Macrae, 2001; MacLeod, 2002; Storm et al., 2006), possible memory impairments for feigners might be due to inhibition of un-practiced information, wherein inhibition is clearly elicited by the strengthened recollection of practiced-target items. Importantly, the difference between total amount of information given by participants in the simulator control group and those in the confessor group resembled the standard memory-undermining effect of feigning amnesia (e.g., Van Oorsouw and Merckelbach, 2004, 2006; Mangiulli et al., 2018b). Note, however, that the simulator control group remembered slightly more Rp – information than confessors. Perhaps, when feigners are not given any specific instructions regarding how to pretend memory loss following a crime, feigning amnesia might be considered as a buffer against forgetting and, at least to some degree, it could increase recollection for such crucial crime-related information (e.g., Mangiulli et al., 2018b; Mangiulli et al., in press).

Finally, with respect to our third hypothesis, while no differences were found between participants in the simulator retrieval-practice group and the confessor group, those in the simulator control group made more errors than confessors. On the one hand, these results might suggest that repeatedly retrieving a specific version of the crime prevents distortion and commission errors during a later recall test when one honestly tries to remember the experience. On the other hand, when individuals come up with an alternative version of the crime (e.g., Van Oorsouw and Merckelbach, 2006) without being specifically instructed regarding how to malingering memory loss, they might be more likely to increase the number of distortions and/or commission errors over time (Chrobak and Zaragoza, 2008; Van Oorsouw and Giesbrecht, 2008; Otgaar and Baker, 2018). Namely, in our study, the retrieval-practice trials might be seen as a form of strategy to feign amnesia. During the attempt to feign memory loss for a crime, simulators RP were forced to omit certain crucial information while simultaneously retrieving other aspects, leading them to poorly remembering those omitted crucial crime-related details. In contrast to simulators RP, however, we do not know which approach simulator controls adopted to come up with their feigned version of the crime. Hence, future research should further examine the strategies adopted by simulators to ascertain to which extent feigning amnesia for a crime might cause different memory outcomes (i.e., omission vs. commission errors and remembering vs. forgetting).

Several limitations of the present work need to be addressed. First, our sample was mainly composed by students, who differ in a myriad of ways from people who perpetrate serious crimes (Schacter, 1986). Although research using laboratory mock crime paradigms are fundamental to increase our knowledge about crime-related amnesia, our findings may have a limited ecological validity. Second, RP + and Rp - information was not counterbalanced across the simulators in the retrieval-practice group. One could argue, indeed, that the lack of counterbalancing across this group does not indicate whether RP + and Rp - information differed on participants’ baseline memorability. However, it should be observed that the valence of both Rp + and Rp - might have differed within simulators in the retrieval-practice group to begin with, leading to a lower correct recollection of Rp - information (e.g., “*I tried to molest a girl*” or “*I put my hands around her neck*”) than Rp + information (e.g., “*My boss scolded me*” or “*I crashed into a tall man*”) during the final recall test (e.g., Barnier et al., 2004; Hauer and Wessel, 2006). Nonetheless, it is unlikely that simulators in the retrieval-practice group could have shown differences between RP + and Rp - information even regardless of the retrieval-practice instruction because RP + and Rp - information pattern observed in this latter group was as opposed to that exhibited by both simulator control and confessor groups. Still, in this study, it remains unclear what information, among all un-practiced items, is most affected by the RIF effect. Arguably, adopting a more controlled memory measure (i.e., cued recall) might enable researchers to draw more specific conclusions on the RIF effect on offenders’ memory impairments. Third, it should be noted here that the after-one-day facilitation effect might have been confounded by the immediate recall, indicating that long-term positive consequences due to the RIF procedure might be difficult to interpret in our study. In a similar vein, although our findings suggest that the RIF effect could potentially occur in crime-related amnesia cases, also the long-term consequences of the RIF effect remain to be determined (e.g., Anderson, 2003; Garcia-Bajos et al., 2009; Storm et al., 2012). For those reasons, therefore, replications of this work are needed.

In closing, the present research might have practical relevance. Many offenders are interviewed by the police about their crimes. Because some of them retrieve their version of the crime in a way to minimize legal and perhaps emotional consequences of their deeds (e.g., Christianson and Merckelbach, 2004), the act of feigning amnesia might lead to strengthening of trivial crime-related details in memory and forgetting of more important facts due to inhibition-based mechanism. Thus, extending RIF to the feigning amnesia paradigm represents a step forward to understanding impaired memory for a crime after feigning amnesia. In addition, even when perpetrators are motivated to plead guilty after having previously feigned amnesia, police investigators should take into account that these offenders might have genuine memory loss for details of their crimes. Of course, because of differences between experimental settings and real-life cases, caution should be exercised when generalizing our findings to actual crime-related amnesia cases. What remains worthwhile, however, is that our study contributes to the understanding of feigners’ memory impairment after simulating amnesia and its relevance in the legal context.

## Data Availability

We will be willing to provide readers with our dataset pertaining to the current study prior to requesting access. Requests to access the datasets should be directed to IM, ivan.mangiulli@maastrichtuniversity.nl.

## Ethics Statement

This study was approved by the Ethical Committee of the Faculty of Psychology and Neuroscience, Maastricht University (ERCPN application – 180_01_06_2017). Written informed consent was obtained from all participants.

## Author Contributions

IM is the main author and coordinator of the study. IM created the idea and methodology of this study with the help and supervision of KvO and MJ, processed the experimental data and performed the analysis assisted by AC, and wrote the manuscript with significant contribution from all the co-authors.

## Conflict of Interest Statement

The authors declare that the research was conducted in the absence of any commercial or financial relationships that could be construed as a potential conflict of interest.

## Footnotes

1 No significant differences were observed among participants on both their positive and negative states before the experimental phase, *F*(2,117) = 0.29, *p* = 0.75, and *F*(2,117) = 0.43, *p* = 0.65.

2 Given that it might be problematic asking to female students to identify themselves with a male offender, we used a mock crime video in *pov* perspective to exclude the potential confounding effect caused by the offenders’ gender.

3 Based on previous research (e.g., Migueles and Garcia-Bajos, 2007; Garcia-Bajos et al., 2009; Pica et al., 2015), a separate group of pilot participants (*N* = 13) was requested to select Rp + and Rp - information.

4 No significant differences were observed among participants belonging to the three groups (simulator RP vs. simulator controls vs. confessors) with respect to their ability to identify themselves with the offender, *F*(2,116) = 1.90, *p* = 0.15.

5 We also conducted an identical mixed model ANOVA with the purpose of ascertaining the facilitation effect by comparing simulator RP to confessor group. Overall, simulators in the retrieval-practice group (*M* = 0.66, *SD* = 0.12) significantly disclosed more RP + information than un-practiced information in the confessor group (*M* = 0.55, *SD* = 0.15), *t*(78) = 3.32, *p* = 0.001, *d* = 0.81.

6 The RIF effect was even found in the comparison between simulators RP and confessors, by running the same mixed model ANOVA. Overall, simulators in the retrieval-practice group (*M* = 0.34, *SD* = 0.12) significantly reported less RP - information than confessors (*M* = 0.55, *SD* = 0.15), *t*(78) = -6.84,*p* = 0.001, *d* = 1.54.

## Appendix A

**Table 1A:** List of practiced and un-practiced information adopted for the retrieval-practice manipulation in the present study.

Practiced information (Rp +)	Un-practiced information (Rp -)
1. I left my apartment	1. I drank vodka at my place
2. I got in my green car	2. I went clubbing
3. I entered my office	3. I drank a cocktail
4. My boss got in the office	4. I drank a shot
5. My boss left me some work-folders	5. I bumped into a guy while I was walking
6. My boss scolded me	6. We started arguing
7. I left the office immediately after	7. I tried to molest a girl
8. I got back home	8. I got drunk
9. I briefly cooked the dinner	9. I vomited in the restroom sink
10. I left my apartment	10. The victim came out of the toilet
11. I bought a beer	11. The victim approached me
12. I set on a chair	12. I pushed the victim against the wall
13. I smoked a cigarette	13. I strongly shook the victim
14. I entered the restroom	14. We had a physical fight
15. I tried to open a toilet	15. I knocked the victim down
16. It was locked up	16. I put my hands around her neck
17. I left the restroom	17. I strangled the victim
18. I crashed into a tall man	18. I moved away from the body
19. I got back in my green car	19. I came back to the toilet
20. I drove toward home	20. I tried to revive the victim

## Appendix B

**Table 1B:** Cued recall task employed for simulators in the retrieval-practice group to retrieve their simulated version of the crime based on Rp + information. Between parentheses are shown missing words of Rp + information that feigners had to fill in.

Well, I do not have a great memory of that day. I remember that I left my **(apartment)** and I got in my **(green car)**. I entered my **(office)** and then my **(boss)** got in. He left me some **(folders)**. Well, I am not really sure but I think he **(scolded)** me. Next, I left the **(office)** immediately and I got back **(home)**. There, I briefly cooked **(the dinner)** and I left my **(apartment)**. Even since then, my memories are not very clear. All seems very confusing and obscure, but still, I remember that I bought a **(beer)** and I sat down on a **(chair)** somewhere. I also smoked a **(cigarette)**. Then, I entered in the **(restroom)**. I tried to open a **(toilet)** but it was **(locked up)**. I left the **(restroom)**, and I crashed into a **(tall man)**. But I am sorry, I do not remember well. Everything is absolutely vague. I think that later I got back in my **(green car)** and I drove toward **(home)**. That is all I remember about that day.
